# *Culicoides* (Diptera: Ceratopogonidae) midges, the vectors of African horse sickness virus – a host/vector contact study in the Niayes area of Senegal

**DOI:** 10.1186/s13071-014-0624-1

**Published:** 2015-01-21

**Authors:** Moussa Fall, Maryam Diarra, Assane G Fall, Thomas Balenghien, Momar T Seck, Jérémy Bouyer, Claire Garros, Geoffrey Gimonneau, Xavier Allène, Iba Mall, Jean-Claude Delécolle, Ignace Rakotoarivony, Mame T Bakhoum, Ange M Dusom, Massouka Ndao, Lassana Konaté, Ousmane Faye, Thierry Baldet

**Affiliations:** ISRA, Laboratoire National de l’Elevage et de Recherches Vétérinaires, Route Front de Terre, Dakar, Senegal; Cirad, UMR CMAEE, F-34398 Montpellier, France; INRA, UMR1309 CMAEE, F-34398 Montpellier, France; UdS, IPPTS, Faculté de Médecine, F-67000 Strasbourg, France; Faculté des Sciences et Techniques, Département de Biologie Animale, Université Cheikh Anta Diop, Dakar, BP 5005 Senegal

**Keywords:** Animal health, Suspected vectors, *Culicoides oxystoma*, *Culicoides imicola*, Population dynamics

## Abstract

**Background:**

African horse sickness (AHS) is an equine disease endemic to Senegal. The African horse sickness virus (AHSV) is transmitted to the mammalian hosts by midges of the *Culicoides* Latreille genus. During the last epizootic outbreak of AHS in Senegal in 2007, 1,169 horses died from this disease entailing an estimated cost of 1.4 million euros. In spite of the serious animal health and economic implications of AHS, very little is known about determinants involved in transmission such as contact between horses and the *Culicoides* species suspected of being its vectors.

**Methods:**

The monthly variation in host/vector contact was determined in the Niayes area, Senegal, an area which was severely affected by the 2007 outbreak of AHS. A horse-baited trap and two suction light traps (OVI type) were set up at each of five sites for three consecutive nights every month for one year.

**Results:**

Of 254,338 *Culicoides* midges collected 209,543 (82.4%) were female and 44,795 (17.6%) male. Nineteen of the 41 species collected were new distribution records for Senegal. This increased the number of described *Culicoides* species found in Senegal to 53. Only 19 species, of the 41 species found in light trap, were collected in the horse-baited trap (23,669 specimens) largely dominated by *Culicoides oxystoma* (22,300 specimens, i.e. 94.2%) followed by *Culicoides imicola* (482 specimens, i.e. 2.0%) and *Culicoides kingi* (446 specimens, i.e. 1.9%).

**Conclusions:**

*Culicoides oxystoma* should be considered as a potential vector of AHSV in the Niayes area of Senegal due to its abundance on horses and its role in the transmission of other *Culicoides*-borne viruses.

## Background

Midges in the genus *Culicoides* Latreille are small biting dipterans (1 to 4 mm in length) that belong to the Ceratopogonidae family. *Culicoides* present a worldwide distribution. Some 1,250 species have been recorded [1], some of which are vectors of viral and parasitic (both protozoan and nematode) pathogens. Their impact is mainly on animal health: in particular, the transmission of virus of two epizootic diseases in horses and ruminants, respectively African horse sickness (AHS) and bluetongue (BT) [2].

AHS is an arboviral disease endemic to sub-Saharan Africa. In the north it is present up to the latitude that links Senegal to Ethiopia. South Africa presents the most southerly distribution. Nine antigenically serotypes are recognized [3]. African horse sickness virus (AHSV) has been responsible for epizootic outbreaks outside of its endemic area in regions previously free of the disease, namely in the Middle East and Asia between 1959 and 1960 [4], in the Maghreb and the Iberian peninsula between 1965–1966 [4], on the Iberian peninsula between 1987 and 1990 [4], in Yemen in 1997 [5], and in the Cape Verde Islands in 1999 [5].

AHS has devastating consequences on susceptible horses with death rates in excess of 90% [6]. Disease control depends on vaccination, isolation or slaughter of infected animals, restrictions on movements of equines and vector control. Only attenuated viral strains are used for vaccination purposes with the drawbacks inherent to the use of live vaccines in addition to the teratogenic effects that prohibit their use on gestating females. Vector control requires the identification of all *Culicoides* species involved in the transmission. However, control against *Culicoides* is very limited in the field due to a lack of knowledge of their bio-ecology and efficient operational methods [7].

The most recent epizootic outbreak of AHS in Senegal in 2007 caused the death of 1,169 horses, with an estimated cost of 1.4 million Euros [8]. Riding centres in the Dakar region were very badly affected with a mortality rate of 8.6% [8]. Virological investigations identified serotype 2 and 7 [8,9] as the responsible viral strains, whereas only serotype 9 had been encountered in Senegal previously [10,11].

Up to 1994, 34 *Culicoides* species were recorded in Senegal [1,12-14]. Amongst these are *Culicoides imicola* Kieffer is a proven vector of AHSV in southern Africa [15,16]. However, similar as to BT in Europe, virus detection in field collected specimens coupled to oral susceptibility results in the laboratory [16-18] infers that susceptibility to AHSV infection is not restricted to a few species but that it is widespread in the genus *Culicoides*.

To identify all potential *Culicoides* vectors in Senegal, we carried out light trap and horse-baited trap collections in an area which experienced outbreaks of AHSV in 2007. Seasonal dynamics of *Culicoides* collected in light traps during these collections was described previously [19], updating the list of species known in Senegal from 34 to 45 [19,20]. In this work we focused on host-baited trap collections with the aim to (i) draw up an inventory of the *Culicoides* species relevant for animal health in the Niayes area of Senegal, (ii) identify *Culicoides* with a host preference for horses and are therefore potential vectors of AHSV and (iii) monitor the seasonal variations in this vector-host contact.

## Methods

### Study area

This study was conducted in the southern part of the Niayes area. The Niayes are depressions between dunes that are liable to flooding during the rainy season. They are located just inland of the coastline of Grande Côte, covering a strip 25 to 30 km wide that follows the coast for 180 km, stretching from Dakar to the mouth of the Senegal River. The climate is of the sub-Canarian tropical type with cool temperatures that vary within a narrow range [21]; the average highest monthly temperature is 27.5°C in Dakar and 28.l °C in Saint-Louis [22]. The presence of the ocean is conducive to a high relative moisture rate ranging from 15% to 90% depending on distance from the sea and time of year. The vegetation in the area is diversified, with a plant cover of less than 50%. Typical vegetation consists of large expanses of household vegetable species (cabbage, potato, tomato, carrot, onion, green beans, salad) and fruit trees (citrus, mango). Dairy and poultry farms are also found. In addition, several riding centres that accommodate exotic breeds of horses are located in this area. Rainfall rarely exceeds 500 mm/year in the south, close to Dakar, and 350 mm/year in the north, close to the Senegal River delta. There are two main seasons in Senegal: the rainy season (from July to October) and the dry season (from November to June), which can be subdivided into a cold dry season (from November to February) and a warm dry season (from March to June). Occult precipitation called the ‘heug’ or mango rains often come during the dry season, and particularly during the cold dry season (December, January and February).

### Collection sites

**Figure 1:**
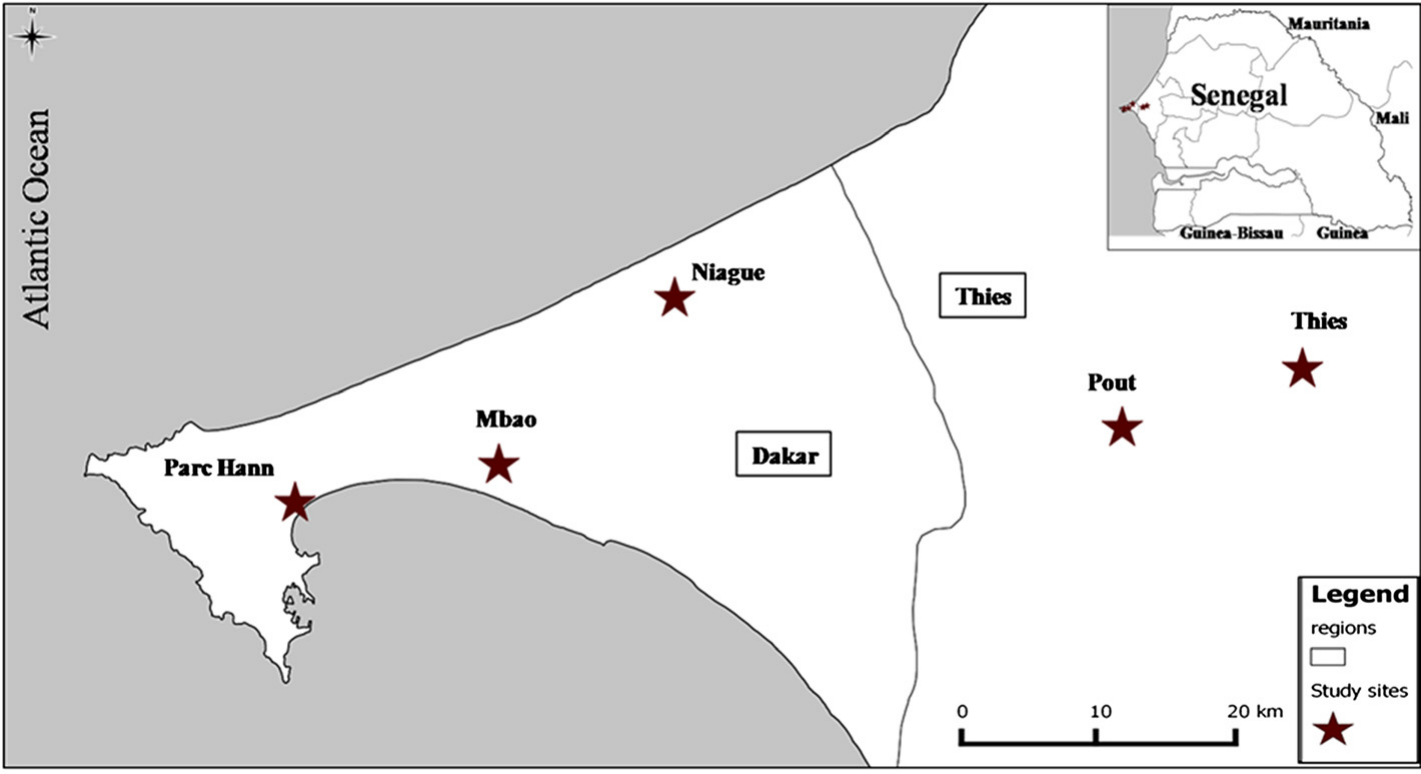
**Location of sampling sites at which light trap and horse-baited trap collections where carried out from July 2011 to October 2012 in the southern part of the Niayes area in Senegal.**

The survey was carried out at five riding centres, described in Diarra *et al*. [20], in the southern part of the Niayes area close to Dakar and Thies (Figure 1). The geographical coordinates and numbers of animals for each site are given by Diarra *et al*. [20].

The reasons for choosing the southern part of the Niayes area and the five sampling sites were its history of repeated AHS outbreaks and its considerable economic potential for stock-breeding in conjunction with many modern farms with exotic breeds established there. Another consideration was the massive mortality among purebred horses during the last epizootic outbreak of AHS in 2007 [8].

### Culicoides trap collections

**Figure 2:**
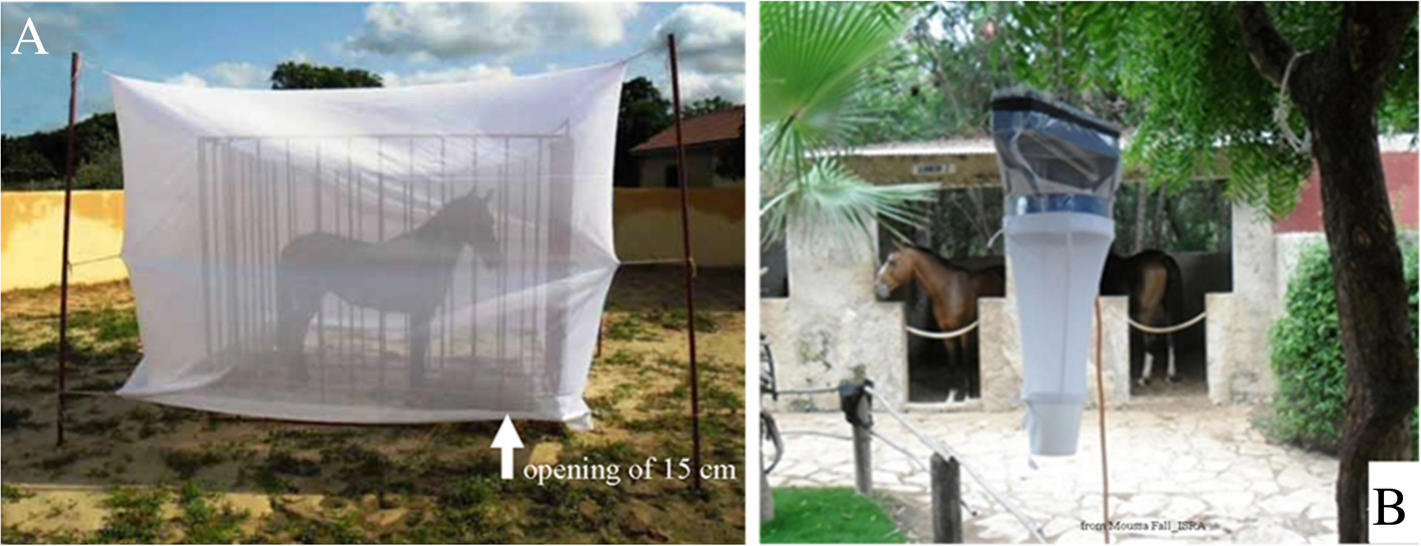
**Description of traps used (A: horse-baited trap; B: light trap) to collect Culicoides at 5 sites in the Niayes area in Senegal from July 2011 to October 2012.**

To assess vector/host contact, *Culicoides* were collected using a horse-baited trap identical to the one used by Fall *et al*. for entomological investigations of mosquitoes’ vectors of West Nile fever [23,24] (Figure 2A). In parallel, two light traps of the OVI (Onderstepoort Veterinary Institute) type were operated to compare host-baited collections with this more widely used *Culicoides* trapping method [25,26] (Figure 2B). The horse-baited trap consists of net boxes (3.5 m × 2.5 m × 2.5 m, with mesh of 1.5 mm × 0.3 mm) with an open space of 15 cm from the ground allowing *Culicoides* to enter, to engorge or not on horse, and avoiding the escape of trapped midges. Both sampling methods were used at each of the five sites for three consecutive nights per month from July 2011 to June 2012 at Parc Hann, Mbao, Thies and Pout sites and from November 2011 to October 2012 at Niague.

The horse-baited trap and the light traps were activated simultaneously, operating from 6 p.m. to 8 a.m. based on the dusk/dawn and night-time activity of most *Culicoides* species. At every site, one of the light traps was placed within 5 m from the door of a loose box containing a horse, while the second was positioned outside the line of vision of the loose box more than 10 m away from the horses. Both light traps were placed at a height of 1.5 to 2 m and sheltered from the wind and any source of artificial light. The horse-baited trap was positioned more than 10 m away from the closest horse housed in a loose box. The three traps were fairly distant from one another (>10 m) so as to minimize possible interactions [27].

The *Culicoides* specimens collected in the light traps were recovered in the morning, strained to separate the insects from the soapy water and transferred to an appropriate labelled jar. Samples were preserved in 90% alcohol and stored in the dark until identified.

As for the horse-baited traps, the animals were fed and watered according to the usual procedures applied at the stable. We used the same five horses, approximately in the same size and weight, during the entire survey: four were females (the male was in Parc Hann) and four were chestnut (the fifth was cream-colored and located at Niague). The *Culicoides* were collected into a collection bottle with a mesh bottom early in the morning using an electric vacuum cleaner for between 10 and 15 minutes. The sampling site and date of capture were indicated on each bottle. These live specimens were killed by freezing (−20°C), sorted and identified the same day before being stored in 90% alcohol in a labelled bottle.

### Identification of the Culicoides

Morphological identification of *Culicoides* species was conducted by examination of the wing pigmentation pattern using a stereomicroscope. For species that are difficult to identify, the specimens were dissected and slide-mounted in accordance with the Wirth and Marston technique for observation under a microscope [28]. Several identification keys were used depending on the species found and their subgenus or group [13,14,29-34]. A count of all the *Culicoides* identified was made by species, gender and, for females, by physiological status. They were stored in 90% alcohol. Samples comprising more than 3 ml of insects were subsampled according to the modified procedure based on Van Ark and Meiswinkel [35].

### Statistical analyses

Monthly abundance of each *Culicoides* species was determined by taking the largest of the catches made on three consecutive nights in every trap. Maximum was preferred to mean because the number of specimens collected can drop very quickly when local weather conditions are sub-optimal. Hence, the maximum of several consecutive collections was considered as the best representation of abundance over a short time period [36]. Capture frequency is the ratio of the number of nights a species was present over the total number of capture nights per site. The engorgement rate for a species is the ratio of engorged females to the total number of females of this species. Engorgement rates were compared between species by means of the Kruskal Wallis test [37]. Monthly abundances were compared between capture methods by performing Pearson’s correlation test.

Diversity of *Culicoides* species by trap and by site was assessed using cumulative monthly abundances by Shannon index and the Simpson-Yule. Between-class (sites) and within-class (trap types) measures were obtained by a centred principal component analysis of the natural logs of the cumulative abundance percentages by species, site and trap type [38,39].

The statistical analyses were all performed with the R programming language at the significance level of 5% [40].

### Ethical approval

This protocol was approved by the Scientific and Technological Advice board of ISRA, Senegal on 22–27 November 2010. The protocol strictly adhered to the usual conditions of animal welfare. Animals used as baits were not no more exposed to *Culicoides* bites than other animals of the private stables in the area. They also received veterinary care during the experiment.

## Results

### Overall diversity

In total, 254,338 specimens of the *Culicoides* genus, of which 209,543 females (82.4%) and 44,795 males (17.6%) were collected using the two sampling methods over the 515 nights of trapping: 181 and 178 for each light trap and 156 for the horse-baited trap (Table 1). Diarra *et al.* [20] listed 24 different species in light trap collections, but 398 specimens remained unsorted and grouped into *Culicoides sp*. After completing the identification, we finally identified at least in light trap and horse-baited trap collections, 19 of which are new distribution records in Senegal (11 from Diarra *et al.* [20] and 8 from this paper). Hence the list of *Culicoides* in Senegal now comprises 53 species (Table 2). The most abundant species captured were *C. oxystoma* (41.4% of overall captures), *C. kingi* (39.0%), *C. imicola* (10.0%), *C. enderleini* (3.9%) and *C. nivosus* (1.4%). The co-dominance of *C. oxystoma* and *C. kingi* is illustrated by a Shannon index of 1.39 (maximum value 3.7) and a Simpson-Yule index of 0.34.

**Table 1:** **Cumulative maximum monthly abundance [number of Culicoides (percentage)] by trap (two light- and horse-baited traps) and by site collected over the year (from July 2011 to October 2012) in the Niayes area of Senegal**

	**Parc_hann**		**Mbao**		**Niague**		**Pout**		**Thies**		**TOTAL (cumulative %)**	**RANK**
	**OVI**	**Horse**	**OVI**	**Horse**	**OVI**	**Horse**	**OVI**	**Horse**	**OVI**	**Horse**		
*C. oxystoma*	5,414 (66.65)	199 (75.95)	22,888 (69.94)	9,290 (97.37)	1,381 (3.11)	8 (2.33)	1,476 (49.27)	12 (35.29)	910 (38.91)	373 (65.10)	41,951 (41.39)	**1**
*C. kingi*	128 (1.58)	3 (1.15)	4,073 (12.45)	104 (1.09)	34,894 (78.56)	148 (43.02)	139 (4.64)		74 (3.16)	3 (0.52)	39,566 (80.43)	**2**
*C. imicola*	261 (3.21)	5 (1.91)	3,718 (11.36)	90 (0.94)	4,588 (10.33)	58 (16.86)	558 (18.62)	17 (50.00)	702 (30.01)	157 (27.40)	10,154 (90.45)	**3**
*C. enderleini*	1,021 (12.57)	4 (1.53)	1,165 (3.56)	40 (0.42)	1,279 (2.88)	3 (0.87)	325 (10.85)	1 (2.94)	152 (6.50)	2 (0.35)	3,992 (94.39)	**4**
*C. nivosus*	451 (5.55)	10 (3.82)	306 (0.94)		267 (0.6)	9 (2.62)	297 (9.91)	2 (5.88)	80 (3.42)	2 (0.35)	1,424 (95.79)	**5**
*C. austeni*	303 (3.73)	27 (10.31)	1 (<0.01)		559 (1.26)	87 (25.29)			6 (0.26)		983 (96.76)	**6**
*C. similis*	134 (1.65)	3 (1.15)	169 (0.52)	3 (0.03)	271 (0.61)		64 (2.14)		48 (2.05)	1 (0.17)	693 (97.44)	**7**
*C. gambiae*	1 (0.01)		34 (0.10)		450 (1.01)	7 (2.03)	3 (0.10)		6 (0.26)		501 (97.94)	**8**
*C. moreli*	4 (0.05)		101 (0.31)	8 (0.08)	215 (0.48)	16 (4.65)	28 (0.93)		9 (0.38)	2 (0.35)	383 (98.32)	**9**
*C. bolitinos*	3 (0.04)		22 (0.07)	4 (0.04)	82 (0.18)	4 (1.16)	9 (0.30)	1 (2.94)	115 (4.92)	21 (3.66)	261 (98.57)	**10**
*C. distinctipennis*	78 (0.96)		64 (0.20)		102 (0.23)	1 (0.29)	10 (0.33)		2 (0.09)		257 (98.83)	**11**
*C. milnei*	217 (2.67)	5 (1.91)	4 (0.01)		30 (0.07)						256 (99.08)	**12**
*C. murphyi*	55 (0.68)		22 (0.07)		114 (0.26)	1 (0.29)	1 (0.03)	1 (2.94)	4 (0.17)		198 (99.27)	**13**
*C. leucostictus*	14 (0.17)	3 (1.15)	68 (0.21)	1 (0.01)	23 (0.05)		50 (1.67)		21 (0.90)		180 (99.45)	**14**
*C. miombo*	1 (0.01)		25 (0.08)		56 (0.13)	1 (0.29)	20 (0.67)		48 (2.05)	7 (1.22)	158 (99.61)	**15**
*C. pseudopallidipennis*			28 (0.09)		16 (0.04)		4 (0.13)		44 (1.88)	4 (0.70)	96 (99.70)	**16**
*C. hortensis*	25 (0.31)	3 (1.15)			33 (0.07)	1 (0.29)					62 (99.76)	**17**
*C. accraensis*	2 (0.02)		1 (<0.01)		2 (<0.01)				38 (1.62)		43 (99.81)	**18**
*C. pycnostictus*	4 (0.05)		13 (0.04)		6 (0.01)		8 (0.27)		10 (0.43)		41 (99.85)	**19**
*C. translucens*					2 (<0.01)				25 (1.07)		27 (99.87)	**20**
*C. pretoriensis*			9 (0.03)						16 (0.68)	1 (0.17)	26 (99.90)	**21**
*C. azerbajdzhanicus*	2 (0.02)				16 (0.04)		2 (0.07)		5 (0.21)		25 (99.92)	**22**
*C. dekeyseri*					20 (0.05)						20 (99.94)	**23**
*C. neavei*			9 (0.03)						1 (0.04)		10 (99.95)	**24**
*C. trifasciellus*					1 (<0.01)		1 (0.03)		8 (0.34)		10 (99.96)	**25**
*C. nevilli*	3 (0.04)		2 (0.01)	1 (0.01)					1 (0.04)		7 (99.97)	**26**
*C. excpectator*									5 (0.21)		5 (99.98)	**27**
*C. nigripennis*									4 (0.17)		4 (99.98)	**28**
*C. ravus*	1 (0.01)				2 (<0.01)		1 (0.03)				4 (99.98)	**29**
*C. robini*	1 (0.01)		1 (<0.01)		1 (<0.01)						3 (99.99)	**30**
*C. quinquelineatus*					2 (<0.01)						2 (99.99)	**31**
*C. vomensis*									2 (0.09)		2 (99.99)	**32**
*C. wansoni*			2 (0.01)								2 (99.99)	**33**
*C. clarkei*									1 (0.04)		1 (99.99)	**34**
*C. congolensis*					1 (<0.01)						1 (99.99)	**35**
*C. dispar*					1 (<0.01)						1 (100.00)	**36**
*C. fulvithorax*									1 (0.04)		1 (100.00)	**37**
*C. punctithorax*									1 (0.04)		1 (100.00)	**38**
*C. sellersi*			1 (<0.01)								1 (100.00)	**39**
*C. vicinus*					1 (<0.01)						1 (100.00)	**40**
*C. yankari*			1 (<0.01)								1 (100.00)	**41**
	**8,123**	**262**	**32,727**	**9,541**	**44,415**	**344**	**2,996**	**34**	**2,339**	**573**	**101, 354**

**Table 2:** **New list of Culicoides species found in Senegal**

**Reported species not found**	**Reported species found**	**Newly recorded species**
*C. africanus* ^‡^ Clastrier	*C. accraensis* ^2^ Carter, Ingram and Macfie	*C. austeni* ^‡1^ Carter, Ingram and Macfie
*C. camicasi* Cornet and Château	*C. clarkei* ^2^ Carter, Ingram and Macfie	*C. azerbajdzhanicus* ^2^ Dzhafarov
*C. chateaui* Cornet	*C. congolensis* ^2^ Clastrier	*C. bolitinos* ^*1^ Meiswinkel
*C. dasyops* Clastrier	*C. dekeyseri* ^2^ Clastrier	*C. hortensis* ^‡1^ Khamala and Kettle
*C. dutoiti* de Meillon	*C. dispar* ^2^ Clastrier	*C. leucostictus* ^1^ Kieffer
*C. grahamii* Austen	*C. distinctipennis* ^1^ Austen	*C. milnei* ^‡1^ Austen
*C. kobae* Cornet and Château	*C. enderleini* ^†1^ Cornet and Brunhes	*C. miombo* ^*1^ Meiswinkel
*C. krameri* ^‡^ Clastrier	*C. expectator* ^2^ Clastrier	*C. murphyi* ^1^ Clastrier and Wirth
*C. micheli* Cornet and Château	*C. fulvithorax* ^2^ Austen	*C. nigripennis* ^2^ Carter, Ingram and Macfie
*C. moucheti* Cornet and Kremer	*C. gambiae* ^1^ Clastrier and Wirth	*C. oxystoma* ^†1^ Kieffer
*C. peretti* Cornet and Château	*C. imicola* ^*1^ Kieffer	*C. pretoriensis* ^1^ Kremer and Nevill
*C. saboyae* Cornet	*C. kingi* ^†1^ Austen	*C. punctithorax* ^2^ Carter, Ingram and Macfie
	*C. moreli* ^‡1^ Clastrier	*C. quinquelineatus* ^‡2^ Goetghebuer
	*C. neavei* ^2^ Austen	*C. sellersi* ^2^ Boorman and Dipeolu
	*C. nevilli* ^†1^ Cornet and Brunhes	*C. translucens* ^2^ Khamala and Kettle
	*C. nivosus* ^1^ de Meillon	*C. trifasciellus* ^2^ Goetghebuer
	*C. pseudopallidipennis* ^*1^ Clastrier	*C. vomensis* ^2^ Boorman and Dipeolu
	*C. pycnostictus* ^2^ Ingram and Macfie	*C. wansoni* ^‡2^ Goetghebuer
	*C. ravus* ^2^ de Meillon	*C. yankari* ^2^ Boorman and Dipeolu
	*C. robini* ^2^ Cornet	
	*C. similis* ^1^ Carter, Ingram and Macfie	
	*C. vicinus* ^2^ Clastrier	

*Species in the Imicola group; ^†^species in the Schultzei group; ^‡^species in the Milnei group.

^1^species captured in horse-baited trap and light trap ^2^species captured only in the light trap.

This update was made on the basis of species collected by light- and horse-baited traps in 2011–2012 in the Niayes area (Diarra et al. [20] and the present study). The individuals identified as C. loxondontis Meiswinkel in Diarra et al. [20] were identified in this paper as C. imicola.

### Culicoides collected on horses

During the overall collection nights, a total of 23,669 specimens were captured in horse-baited traps (9.3% of overall captures) as against a total of 230,670 specimens in the light traps (accounting for 90.7% of overall captures). Only 19 of the 41 species found in light traps were found in horse-baited trap (Table 2). No species was found only in horse-baited trap.

*Culicoides oxystoma* was the dominant species on horse (22,302 individuals; 94.2% of overall captures). The three most abundant species, *C. oxystoma*, *C. imicola* and *C. kingi*, represented 98.2% of collections (23,239 individuals) and the eight most abundant, which included *C. enderleini* and *C. bolitinos* (Table 3), 99.5% of collections.

**Table 3:** **Mean cumulative maximum monthly abundance (maximum) - frequency of Culicoides collected in the 5 study sites (N = No. collections) in horse-baited traps (from July 2011 to October 2012) in the Niayes area of Senegal**

	**Parc Hann (N = 36)**	**Mbao (N = 36)**	**Niague (N = 36)**	**Pout (N = 34)**	**Thies (N = 36)**	**Total (N = 178)**
*C. oxystoma*	16.58 (105) - 100.0	774.17 (8,340) - 100.0	0.67 - 58.3	1.20 (4) - 70.0	31.08 (149) - 75.0	**170.38 (8,502) - 100.0**
*C. imicola*	0.42 (3) - 25.0	7.50 (27) - 75.0	4.58 (21) - 75.0	1.70 (5) - 80.0	13.08 (69) - 83.3	**5.59 (80) - 100.0**
*C. kingi*	0.25 (3) – 8.3	8.67 (47) - 83.3	11.17 (50) - 100.0	0	0.25 (2) - 16.7	**4.21 (58) - 100.0**
*C. enderleini*	0.33 (2) – 16.7	3.33 (35) - 33.3	0.17 (1) - 16.7	0.10 (1) - 10.0	0.17 (1) - 16.7	**0.84 (35) - 66.7**
*C. austeni*	2.25 (12) - 50.0	0	0.50 (4) - 16.7	0	0	**0.57 (12) - 58.3**
*C. bolitinos*	0	0.33 (2) - 25.0	0.17 (1) - 16.7	0.10 (1) - 10.0	1.75 (11) - 41.7	**0.48 (11) - 58.3**
*C. moreli*	0	0.67 (3) - 41.7	1.33 (8) - 25.0	0	0.17 (1) - 16.7	**0.45 (10) - 50.0**
*C. nivosus*	0.83 (4) - 58.3	0	0.75 (2) - 41.7	0.20 (1) - 20.0	0.17 (1) - 16.7	**0.40 (4) - 91.7**
**Total**	**20.67 (118) - 100.0**	**794.67 (8,430) - 100.0**	**19.33 (73) - 100.0**	**3.30 (9) - 75.0**	**46.67 (224) - 91.7**	

**Figure 3:**
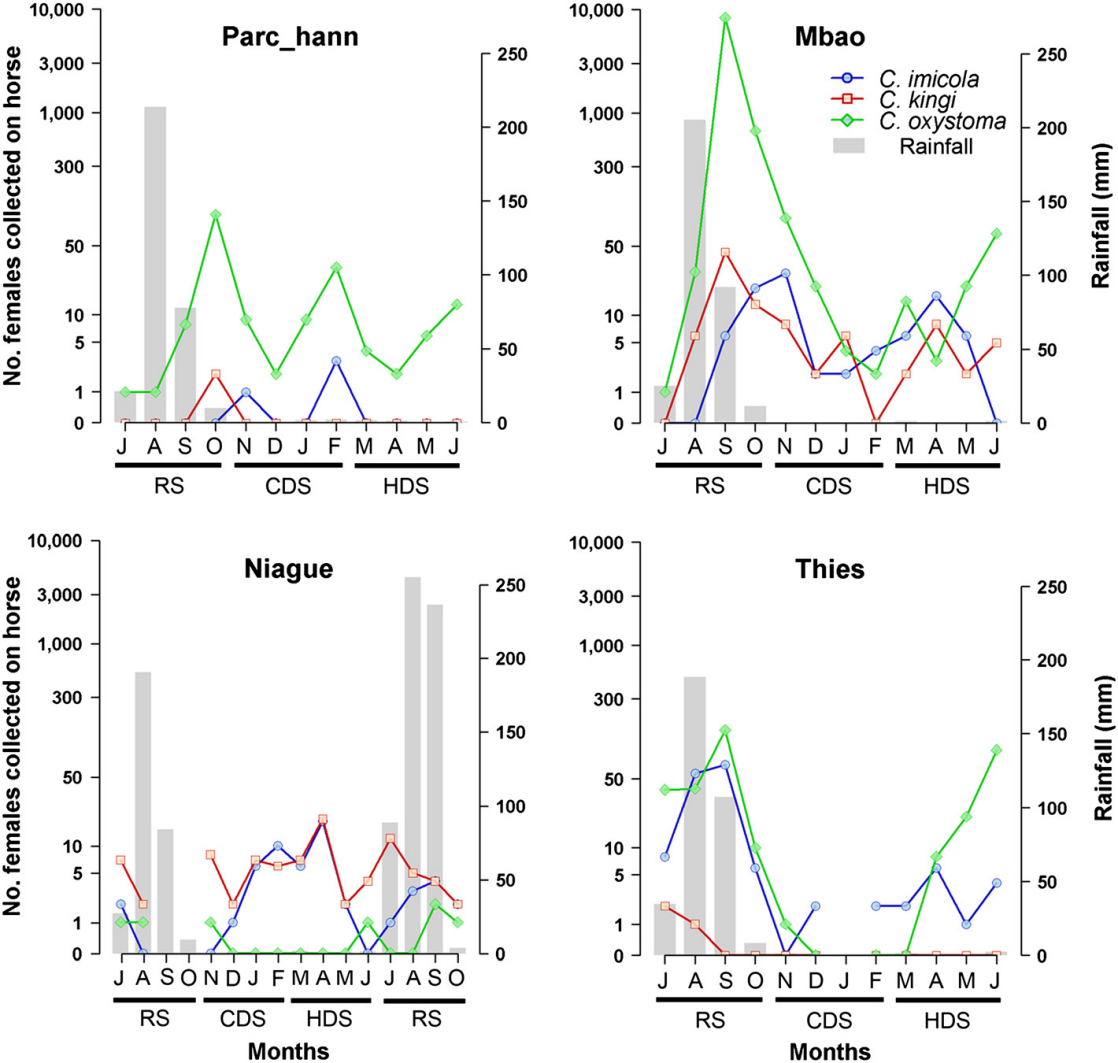
**Dynamics of monthly abundances of females for the three main species (C. oxystoma, C. imicola and C. kingi) in the horse-baited trap by season at 5 sites of the Niayes area in Senegal. **(RS = rainy season; CDS = cold dry season; HDS = hot dry season). NB: a log10 (n + 1) transformation was applied to the abundance data; the Pout site was not plotted due to the low abundances observed.

Overall, biting rates on horses were high at Mbao, medium at Hann, Niague and Thies and relatively low at Pout (Table 3). For *C. oxystoma*, the mean value at Mbao was 772 females/trap/night as compared to 15 or 30 females/trap/night at the Hann Pony Club and at Thies, and close to 0 at Niague and Pout. The highest attack rates on horses were observed in the second half of the rainy season (September and October) at Parc Hann, Mbao and Thies for *C. oxystoma, C. imicola* and *C. kingi* (Figure 3). At these three sites, the primary peak of attack rates was accompanied by a secondary peak during the hot dry season before the rains set in. These species had low abundances during the cold dry season (November to February). In contrast, at Niague, *C. kingi* appeared to be attracted by the horse all year round, and *C. imicola* during the dry seasons (Figure 3).

All engorged females found in the horse-baited traps belong to the Imicola, Schultzei and Milnei groups (Table 4), with an overall engorgement rate of 36.7%. Differences in engorgement rates between species were significant (Kruskal Wallis test, p = 0.03). The engorgement rates may be classified in three groups: the species with an engorgement rate of more than 75% that include *C. bolitinos*, a proven vector of AHSV in South Africa, the species whose engorgement rate ranges between 50 and 75% that include *C. imicola* the main vector of AHSV in Africa, and those whose engorgement rate is less than 50% that include the species in the Schultzei group: *C. oxystoma*, *C. enderleini* and *C. kingi*. Females of *C. gambiae*, *C. leucostictus*, *C. murphyi*, *C. nevilli*, *C. nivosus* and *C. similis* species which were rarely collected in the horse-baited trap were found not to be engorged.

**Table 4:** **Overall engorgement rate of female Culicoides caught in the horse-baited trap at the five sampling sites (from July 2011 to October 2012) in the Niayes area of Senegal**

**Species**	**Number of engorged females [total females]**	**Engorgement rate (%)**
*C. hortensis*‡	4 [4]	100.0
*C. milnei*‡	4 [5]	80.0
C. bolitinos*	28 [36]	77.8
C. miombo*	6 [8]	75.0
C. imicola*	299 [465]	64.5
C. pseudopallidipennis*	3 [5]	60.0
*C. austeni* ^‡^	85 [156]	54.5
*C. moreli* ^‡^	16 [44]	36.4
*C. oxystoma* ^†^	7,943 [21,941]	36.2
*C. enderleini* ^†^	20 [99]	20.2
*C. kingi* ^†^	62 [308]	20.1

*Species in the Imicola group; ^†^species in the Schultzei group; ^‡^species in the Milnei group.

Are listed only species from which at least one female was found engorged.

### Comparison with light trap collections

A total of 127,197 *Culicoides* was collected by the light trap located <5 m from the horses, whereas the light trap located >10 m from the horses collected 103,473 *Culicoides*. The total number of *Culicoides* by species was highly correlated between traps (r = 0.95, p < 0.001), the nine most abundant species (98% of the total collection for both traps) being the same. The three or four most abundant species (*C. oxystoma* and/or *C. kingi*, *C. imicola* and *C. enderleini*) presented the same rank at each site, with the only exception of Mbao where *C. kingi* and *C. imicola* were at the rank 2 and 3 in the light trap <5 m from the horses (12.8% and 9.2% of the total collection) and at the rank 3 and 2 in the light trap >10 m from the horses (15.2% and 24.1%). The differences of proportional representation were low between both light traps for the four most abundant species (mean = 4%, N = 20), with only three differences > 4%: the differences were 33% between collections of the light trap < 5 m and of the light trap > 10 m for *C. oxystoma* at Mbao, but −10% for this species at Hann, and −18% for *C. kingi* at Niague. Moreover the monthly abundances estimated with the light trap < 5 m and the light trap > 10 m were highly correlated (r = 0.73, p < 0.001). Considering thus that both light traps gave comparable results, this data was grouped for the remainder of the analyses, while maintaining the maximum abundance observed per species, site and month.

We compared horse-baited trap collections to light trap collections using two complementary approaches: 1) a principal component analysis (PCA) using between and within-classes decomposition to quantify relative influence of both sites and trap types on *Culicoides* diversity as a whole, and 2) correlations between horse-baited and light trap collections by species.

**Figure 4:**
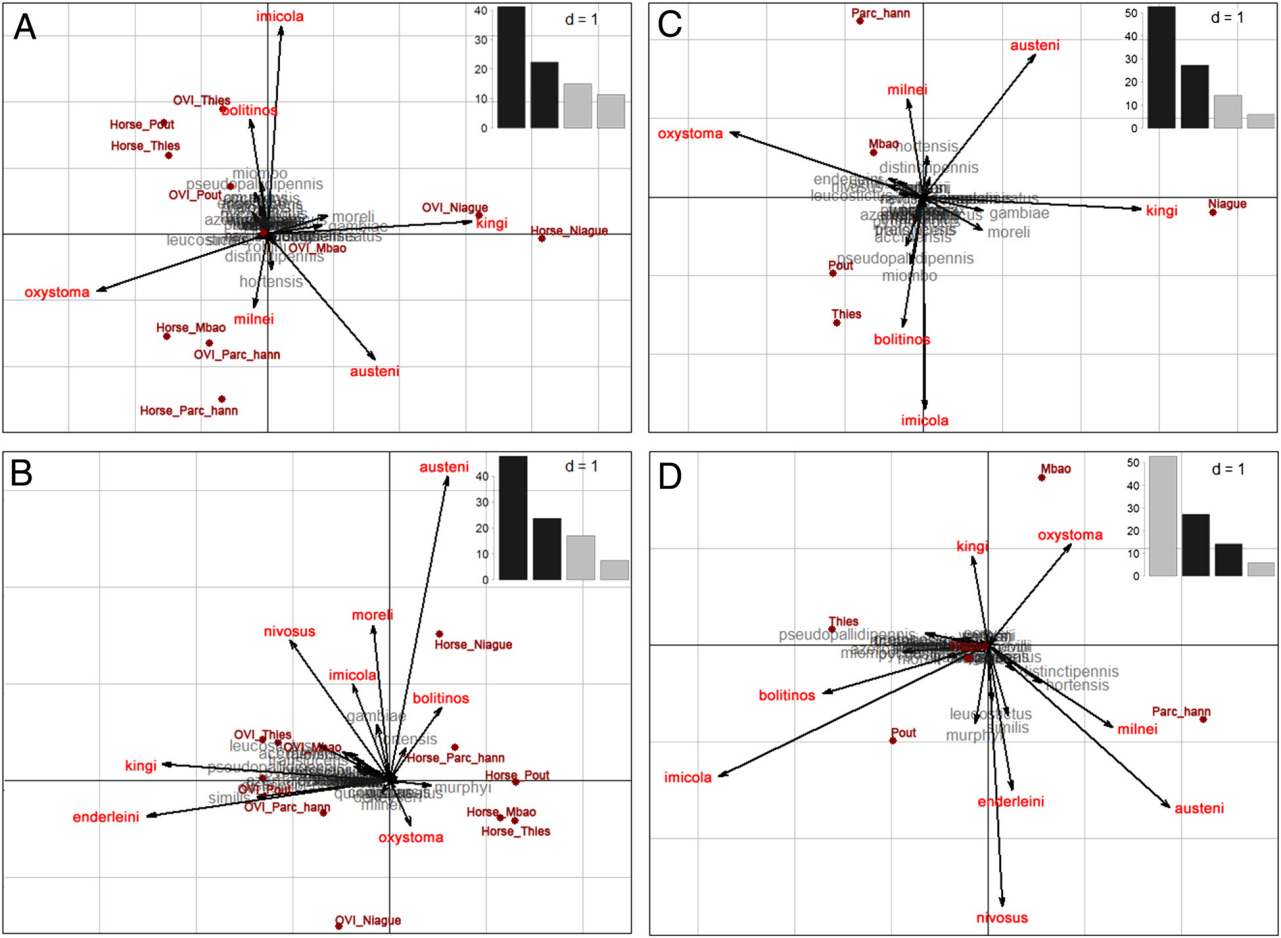
**Diversity of the Culicoides captured by site (5 sites in the Niayes area of Senegal) and trap (light- and horse-baited trap) from July 2011 to October 2012. A**: Centred principal component analysis (PCA) performed on the natural logs of the cumulative percentages of abundance by species, site and type of trap (axis 1/axis 2). **B**: PCA within-class analysis (axis 1/axis 2). **C** and **D**: PCA between-class analysis (axis 1/axis 2 and axis 2/axis 3).

In a first step, centred PCA was performed on the natural logs of the percentages of cumulative abundance by species, site and type of trap. The four first axes represented 89.8% of total variance (Figure 4A respectively 41.3%, 63.2% and 78.6% for each of the first three axes). Between-class (between sites) variance explained 74.0% (as against 44.9% predicted by a permutation test, p < 0.01) of the PCA variance, while within-class (between trap types) accounted for 26.0%, indicating that the site effect was much more predominant in *Culicoides* diversity than the trap effect. This result was illustrated by the resemblance of Figure 4A (PCA) and Figure 4C (PCA with a maximisation of the variance between sites) with the opposition on axis 1 between the Niague site characterised by *C. kingi* and the other sites characterised by *C. oxystoma*, and on axis 2 between Parc Hann and Mbao sites characterised by *C. austeni* and *C. milnei* and Pout and Thies sites characterised by *C. imicola* and *C. bolitinos*. Figure 4D illustrated the opposition between Mbao characterised by both *C. oxystoma* and *C. kingi*, and Parc Hann and Pout characterised by *C. enderleini* and *C. nivosus* on axis 3. Indeed, in Parc Hann, *C. oxystoma* was dominant (66.7% of light trap catches) and was associated with *C. enderleini* (12.6%), in Mbao *C. oxystoma* was predominant (69.9%) in association with *C. kingi* (12.5%) and *C. imicola* (11.4%), in Niague the prevalent species was *C. kingi* (78.6%) in association with *C. imicola* (10.3%), in Pout *C. oxystoma* was dominant (49.3%) alongside *C. imicola* (18.6%) and *C. enderleini* (10.9%) and in Thies *C. oxystoma* was predominant (38.9%) followed by *C. imicola* (30.0%). Hence there was a west-to-east gradient of increasing proportional representation of *C. imicola* (from 3.2 to 30.0%) and of decreasing proportional representation of *C. oxystoma* (from 66.7 to 38.9%, with the exception of Niague where *C. oxystoma* was less represented [3.1%]). Within-class (between trap types) analysis showed differences in catch diversity as between light and baited traps (Figure 4B). This structure was mainly attributable to the species *C. kingi* and *C. enderleini*, which were chiefly captured by light traps (total cumulative monthly abundance is respectively 39,308 and 3,942 for the light trap as against 258 and 50 for the baited trap).

**Figure 5:**
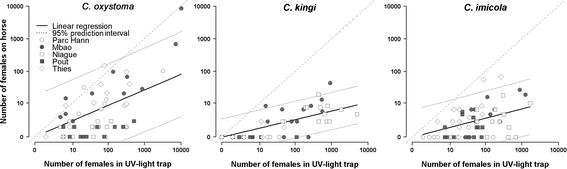
**Correlation of monthly abundances for females collected with light traps and those captured in horse-baited traps from July 2011 to October 2012 at 5 sites in the Niayes area of Senegal.** The 95% prediction interval corresponds to interval in which future observations will fall, with a 95% probability, assuming that future observations have the same error variance as those used for fitting (observation data).

In a second step, correlations were assessed between horse-baited and light trap collections. Monthly abundances of female *C. oxystoma* captured in the light traps were correlated with those collected on the horse bait (Figure 5) at the Parc Hann, Mbao and Thies sites, but not for the other two sites where hardly any of this species were captured on the horse. For those three sites, light trap captured more *Culicoides* by a factor of 1.57 with a log_10_ scale (log_10_(No. *Culicoides* on horse + 1) × 1.57 = log_10_(No. *Culicoides* in light trap + 1); R^2^ = 85.4; p < 0.001) than horse baited trap. *Culicoides kingi* was captured on the horse bait in Mbao and Niague alone. At these two sites (Figure 5), the light trap considerably collected more *Culicoides* by a factor of 3.82 with a log_10_ scale (R^2^ = 74.1; p < 0.001) than horse baited trap. Light trap catches of *C. imicola* were correlated with the horse-baited estimated abundances at the Mbao and Thies sites (Figure 5), where the light trap collections were greater by a factor of 2.41 with a log_10_ scale (R^2^ = 75.6; p < 0.001). There was no correlation between horse-baited and light trap collections for *C. enderleini* or *C. nivosus*.

## Discussion

The *Culicoides* inventory conducted during these horse-baited and light trap surveys in a relatively limited area in the southern part of the Niayes area provided an updated list of *Culicoides* for Senegal comprising 53 species and, more significantly, established the presence and abundance of *C. oxystoma* on horse-baited trap. This species’ distribution was previously confined to the Eastern and Australasian Regions [30,41] and was during this survey identified for the first time in the Afro-tropical Region [19]. In Senegal, there are now at least four species that belong to the Schultzei group: *C. enderleini*, *C. kingi*, *C. nevilli*, and *C. oxystoma*. The presence of a number of other species in the Imicola group such as *C. bolitinos* and *C. miombo* and in the Milnei group such as *C. austeni*, *C. hortensis*, *C. milnei*, *C. quinquelineatus* and *C. wansoni* was also recorded for the first time in Senegal (some of these species were already mentioned by Diarra *et al.* [20]). Twelve species previously captured in other bioclimatic areas of Senegal [12,14] were not detected during this survey.

*Culicoides oxystoma* was the most abundant species associated with horses in the Niayes area, which is known for past outbreaks of AHS [11], and where the latest AHS epizootic in Senegal in 2007 caused considerable losses [8,42]. This raises questions about its role in transmitting the AHSV in Senegal. Its competence against AHSV has never been determined. *Culicoides oxystoma* is known to be involved in the transmission of bovine arboviruses such as Akabane in Japan [43,44]. It is a suspected vector of epizootic haemorrhagic disease virus in Israel [45] and a potential vector of the bluetongue virus (BTV) in India [46].

To a lesser extent, horses were also attacked by *C. imicola* in Mbao, Niague and Thies, with *C. kingi* in Mbao and Niague, with *C. enderleini* in Mbao, with *C. austeni* in Parc Hann, and by *C. bolitinos* in Thies. *Culicoides imicola* is a proven vector of AHSV in South Africa [47] and probably in the Maghreb (Morocco) and the Iberian peninsula (Spain and Portugal) [48]. It is also the main vector of BTV in South Africa, in the Maghreb and in Southern Europe [49]. The role of *C. kingi* and of *C. enderleini* as vectors of AHSV is not clearly established. Field-collected specimens of *C. kingi* in Sudan have been found to be infected by epizootic haemorrhagic disease virus [50]. *Culicoides enderleini* is a potential vector for BTV in South Africa [47,51] and experimental infections conducted in the laboratory have shown that AHSV could be isolated in field specimens of this species 10 days after feeding on a virus-infected blood meal [52]. *Culicoides bolitinos*, the second proven vector for AHSV in South Africa [16], was rarely found at our sampling sites. It is difficult to conclude if this species may play a major role in the transmission of AHSV in Senegal because only a few sites were sampled. This species is mostly found in sub-tropical regions with a humid, cool climate like the coast and mountains of South Africa and locally closely linked with the presence of bovids. Indeed, the coprophilous larvae of *C. bolitinos* develop preferentially on cattle, buffalo and wildebeest dung [16]. However, molecular studies should be conducted on the rare individuals of *C. bolitinos* found in Senegal to ascertain their specific status in view of the many morphological variations observed on these specimens. It is interesting to note that *C. bolitinos* is morphologically similar to *C. brevitarsis*, a species whose coprophilous larvae also develop on cattle dung [53] and whose distribution in the Eastern and Australasian Regions is identical to that of *C. oxystoma*.

In tropical areas, high numbers of *Culicoides* are generally observed at the end of the rainy season, with significant decreases in population during the dry season, in connection with rainfall, relative moisture and temperature which impact both the productivity of larval habitats and the activity of adults [49,54]. The same trend was found in Senegal where the highest abundances of *C. oxystoma* were observed at the end of the rainy season (September and October) and, to a lesser extent, at the end of the dry season (June). These seasonal dynamics for the dominant species in horse-baited traps was highly correlated with that in light traps [20]. These two peaks of abundance coincided with the periods of emergence and expansion of cases of AHS during the 2007 outbreak [42].

The dynamics for the three most abundant species on horses differ according to the site. Between-site variations in the dynamics of *C. oxystoma* may depend on local ecological conditions, in particular the productivity of the available larval habitats [55]. Indeed, the Mbao and Hann sites are respectively located close to a river and a marsh whereas in Thies flooding of larval habitats and their productiveness may be more dependent on rainfall alone. Some of the species in the Schultzei group are regularly found along streams and drainage canals and in mud containing little organic matter [56]. The larvae of *C. oxystoma* can develop both in mud and sand. This may explain the overall abundance of *C. oxystoma* in the Niayes area (depressions between dunes that are liable to flooding in the rainy season). Studies out in India have shown that *C. oxystoma* is a euryhaline species that is present all year round in the Sagar Island estuary in India [57,58]. *Culicoides kingi*, a species typical of brackish environments [13], is the dominant species at the Niague site located close to Lake Retba (the pink lake) where salt is harvested. At this location, the species’ maximum abundance occurs at the end of the dry season (June) and diminishes with rain when the salt concentration in breeding sites may drop creating conditions that are less conducive to the development of this species. This phenomenon is documented for *Anopheles melas* (Diptera: Culicidae), a mosquito species in the *Anopheles gambiae* complex whose halophilic larvae develop on West African lagoons [59]. As for *C. imicola*, *C. kingi* is relatively abundant at Niague and Mbao with essentially year round activity but its larval ecology is poorly known and deserves investigation, particularly in Senegal.

The high engorgement rates of the specimens belonging to the Imicola group, including *C. bolitinos* and *C. imicola*, confirmed the trophic preference of these two species for horses [60,61] and the probable role of *C. imicola* in the transmission of AHSV in Senegal in relation to its abundance and vector status in South Africa [15,47]. However, the average below engorgement rates on horses of the main species in the Schultzei group (*C. oxystoma*, *C. kingi* and *C. enderleini*) may be offset by their abundances, in particular as regards *C. oxystoma*, if it is proven that these species are competent vectors of AHSV.

It is established that light trap collections do not assess biting rates on animals accurately [62,63]. Nevertheless, both methods gave the same picture of *Culicoides* diversity as a whole, as highlighted by the decomposition of PCA variance into between-class (sites) and within-class (traps). This PCA was carried out using the proportional representation rather than the abundance to avoid a “size effect” due to important difference of abundance between sites and between traps. Using abundance in PCA, data were structured mainly along a single axis, highlighting the opposition for *C. kingi* and *C. enderleini* between light traps with high abundance and horse-baited traps with low abundances. This structure driven by *C. kingi* and *C. enderleini* was also found in the within-classes analysis.

The horse-baited trap collected fewer species and is therefore more appropriate for investigating host/vector contact. The difference is reflected by the over-representation of species such as *C. oxystoma* (1.57 in a log_10_ scale), *C. kingi* (3.82 in a log_10_ scale) and *C. imicola* (2.41 in a log_10_ scale) in the light traps compared to the horse-baited traps. Determining such as correction factors is crucial to rescale wide-scale abundance maps, established with light traps as the most convenient trapping method, to probable biting rates which could be used in transmission models.

## Conclusion

This study allows an update of list of *Culicoides* species of veterinary interest in Senegal (53 species to date). *Culicoides oxystoma*, and to a lesser extent *C. kingi* and *C. enderleini*, were furthermore identified as potential vectors of AHSV in the Niayes area of Senegal additionally to the vector *C. imicola* due to their abundance and aggressiveness on horses, as well as their role as vectors for transmitting other animal viral diseases such as Akabane and Epizootic haemorrhagic disease viruses. These preliminary results should however be corroborated by further studies, both in the field and in the laboratory, so as to better specify their bio-ecology, in particular their larval biotope, and their vector competence, and gain a better understanding of their role in the epidemiology of AHS in Senegal.
